# Supplementary material for: Collaborative intelligence in AI: Evaluating the performance of a council of AIs on the USMLE
Source: PLOS Digit Health. 2025 Oct 9;4(10):e0000787. doi: 10.1371/journal.pdig.0000787 (PMC12510544; doi:10.1371/journal.pdig.0000787)
Supplement: S2 File — (ZIP) [file pdig.0000787.s002.zip › S2 Data Raw data files containing unprocessed question inputs and LLM outputs including transcripts of deliberations/STEP3_Question-125-1_Type-MC-NJ.docx]

**Facilitator** **[Round 1]:** A 28-year-old man comes to the office because he would like to quit smoking cigarettes. He says, ""I cannot go more than a day without cigarettes because I get so anxious and irritable. I even wake up at night to smoke."" The patient began smoking cigarettes at age 12 years; he was smoking two packs of cigarettes daily by age 17 years, and he has not curtailed his use since that time. He does not drink alcoholic beverages or use illicit drugs. Medical history is remarkable for childhood febrile seizures. He takes no medications. Vital signs are normal. The patient is interested in using a nicotine patch and bupropion therapy because he says his friend found the treatments to be helpful. In combining these two therapies, this patient is at greatest risk for which of the following? (A) Cold intolerance (B) Hypertension (C) Polyuria (D) Renal insufficiency (E) The patient is at no increased risk

**AI_C:** RESTATING THE QUESTION: This question is asking about the possible risks that this 28-year-old chronic smoker might experience by using a smoking cessation program involving a nicotine patch and bupropion, which are medications used to reduce craving and withdrawal symptoms associated with nicotine dependence. PLANNING: The options provided include cold intolerance, hypertension, polyuria, renal insufficiency, and no increased risk. Cold intolerance isn't typically associated with either nicotine patches or bupropion. Polyuria and renal insufficiency might occur if there are pre-existing renal diseases which is not stated in the case information. Therefore, it might be best to focus on the remaining risk: hypertension. CLINICAL REASONING: The nicotine patch represents a form of nicotine replacement therapy and is known to stimulate the sympathetic nervous system like nicotine from cigarettes, increasing heart rate and blood pressure due to vasoconstriction. Bupropion, an antidepressant, is also used as a smoking cessation aid. It's known to potentially cause an increase in blood pressure, as a rare side effect especially when administered with nicotine patch. Therefore, by combining these two therapies, there could be a compounding of these hypertensive effects. CONCLUDING: Considering the patient's smoking history and current interest in this combination treatment plan, the potential risk for hypertension should be assessed and monitored closely as both bupropion and nicotine replacement therapy may contribute to raising blood pressure levels. RESPONSE: On the available options, (B) Hypertension would present the greatest potential risk to the patient from starting a combined smoking cessation therapy of a nicotine patch and bupropion.

**AI_B:** RESTATING THE QUESTION: A 28-year-old man who has been a significant smoker for years is planning to quit. He is interested in using a nicotine patch and bupropion therapy to aid in smoking cessation. The question here is, what would be the greatest risk that he could develop if these two treatments are combined? PLANNING: In thinking through this question, the main assumption is that a combination of nicotine replacement therapy (using a patch) and bupropion, a non-nicotine medication, may have some potential side effect(s) for this patient, who is a long-time and heavy smoker. Given his history, it's also important to consider his heightened risk for cardiovascular problems. Questioning this assumption could lead to exploring how each drug may individually affect the patient, and whether these effects could be amplified when combined. This would involve understanding the pharmacology of both nicotine and bupropion. CLINICAL REASONING: Bupropion is used for smoking cessation and depression. Bupropion lowers seizure threshold and may lead to increased risks of seizures, especially in patients with pre-existing conditions that are predisposing. Therefore, in view of the patient's history of childhood febrile seizures, the use of bupropion may not be advisable. However, upon carefully examining the answer choices offered, 'increased risk of seizures' is not one of them. We therefore need to shift focus. Among the options given, hypertension stands out as a potential risk due to the use of both the nicotine patch and bupropion. Nicotine can induce vasoconstriction and increased blood pressure, while bupropion inhibition of norepinephrine and dopamine reuptake could lead to an increase in sympathetic activity and thereby blood pressure. The combination might thus heighten this risk. CONCLUDING: The patient's clinical profile and the potential additive effects of nicotine patch and bupropion therapy could increase this patient's risk for hypertension. Clinical caution and monitoring for this outcome would be advisable. RESPONSE: B. The patient is at greatest risk for hypertension.

**AI_A:** 1) RESTATING THE QUESTION: A 28-year-old man who has been a heavy smoker since age 17, without any reported history of alcohol or illicit drug use, is considering using a combination of a nicotine patch and bupropion to help him quit smoking. These forms of Nicotine Replacement Therapy and medication respectively, are commonly used to assist individuals in managing cravings and symptoms of nicotine withdrawal. He has a medical history of febrile seizures during childhood but currently takes no medications and his vital signs are normal. The question is asking what is the greatest risk factor associated with such a combination of therapy? 2) PLANNING: We know that both bupropion, an antidepressant also used for smoking cessation, and nicotine patches, can produce some side effects. For example, Bupropion can lead to an increase in blood pressure (hypertension), while the nicotine patch can sometimes cause skin reactions or cold intolerance. However, these treatments are typically well tolerated and do not pose significant risk for people with normal health profiles. The question here is whether the combination of the two would amplify any negative effects. 3) CLINICAL REASONING: Given the patient's long history of heavy smoking and his anxiety about quitting, it is likely that his nicotine dependence is high. Both nicotine patches and bupropion could help manage the symptoms of nicotine withdrawal, such as irritability and cravings. However, both of these substances could potentially raise blood pressure, especially in a person with an existing high nicotine dependence and associated autonomous nervous system dysregulation. Therefore, the main risk with this combination would likely be the consequent increase in blood pressure, or hypertension. 4) CONCLUDING: Comparatively looking into the other options, while nicotine can impact temperature regulation, it is unlikely to cause cold intolerance in a significant manner. Nicotine and bupropion do not typically cause polyuria or renal insufficiency. On the other hand, stressing on the assertive aspect that "the patient is at no increased risk" is not entirely accurate considering the potential for increased blood pressure with this combination. 5) RESPONSE: Therefore, combining nicotine patches with bupropion does not pose significant risks for many of the options provided, but there is an increased risk for hypertension (B), making this the most likely answer for this patient.

**AI_E:** 1) RESTATING THE QUESTION: The query at hand pertains to a 28-year-old man who is a long-term heavy smoker wanting to quit the habit using a combined therapy of nicotine patch and bupropion. The issue we need to consider here is what potential risk this combined therapy might impose on the patient's health, based on the options provided. 2) PLANNING: In this scenario, the assumptions appear to be that the use of a nicotine patch and bupropion could lead to potential side effects or risks. It would be essential to analyze each potential effect listed, such as cold intolerance, hypertension, polyuria, renal insufficiency or the absence of any significant risk increase. 3) CLINICAL REASONING: Starting with the first alternative, cold intolerance is generally not a side effect associated with either nicotine replacement therapy or bupropion. Moving onto hypertension, while nicotine can increase blood pressure; the use of nicotine patches usually does not significantly elevate blood pressure. Bupropion is also not generally associated with hypertension. Regarding polyuria or increased urine production, it is not a known typical side effect of either treatment. Analysing renal insufficiency, neither nicotine replacement nor bupropion is known to adversely affect kidney function significantly. Finally, while every medication carries some risks and potential side effects, in this case, the combination of nicotine replacement therapy with bupropion is generally well tolerated and is not typically associated with the specific risks listed. 4) CONCLUDING: It would thus seem reasonable to deduce that the health concerns raised: cold intolerance, hypertension, polyuria and renal insufficiency may not be the most pertinent risks for the patient to consider when deciding to use a combination therapy of nicotine patch and bupropion to aid in smoking cessation. It is, of course, important that the patient fully understands all the potential, albeit rare, side effects of the proposed treatment and is monitored during his quit smoking journey. 5) RESPONSE: Therefore, based on the evidence at hand, it would seem that the patient, while taking precautions, is at no significant increased risk (Choice E) of the aforementioned potential hazards when using a combined therapy of nicotine patch and bupropion. It's worth noting, however, smoking cessation can often lead to withdrawal symptoms, including heightened anxiety and irritability, which can be particularly challenging in the early stages of quitting. As such, these are aspects that will need to be managed with a combination of pharmacological and behavioral interventions.

**AI_D:** (1) RESTATING THE QUESTION: You're asking about a 28-year-old man who wants to quit smoking. He started smoking at a young age and has since been a heavy smoker, reaching two packs a day by 17 years old. He experiences withdrawal symptoms when he tries to quit, which include anxiety and irritability. He doesn't use alcohol or illicit drugs, has a history of febrile seizures from childhood, and is not on any medication. His vital signs are normal. He is considering using a combination of a nicotine patch and bupropion therapy to help quit smoking, as a friend found them helpful. We're considering what kind of increased risk he faces if he starts using these treatments. (2) PLANNING: The symptoms he describes when he attempts to stop smoking such as anxiety and irritability are suggestive of nicotine withdrawal. Nicotine replacement therapy (the patch) and bupropion are both common and effective treatments for nicotine dependence. Considering their known side effects: - Nicotine patches could potentially cause local skin irritation, insomnia, vivid dreams, and in some cases, cardiovascular issues due to the constant supply of nicotine, especially in heavy smokers. - Bupropion, on the other hand, is an atypical antidepressant and smoking cessation aid which can cause several side effects including dry mouth, nausea, insomnia, tremor, excessive sweating and in rare cases, seizures. It is noteworthy that the patient has a history of childhood febrile seizures which might increase the risk of this rare side effect. So the risk could be hypertension from nicotine patch or seizures from bupropion. (3) CLINICAL REASONING: However, the nicotine patch would transition him from smoking, which provides an irregular supply of nicotine, to the patch, which provides a steady supply of nicotine throughout the day, thus helping mitigate the symptoms of withdrawal. As such, while cardiovascular issues are a potential side effect, it would not necessarily result in hypertension. On the other hand, the bupropion could increase the patient's risk of seizures, particularly due to his history of childhood febrile seizures. Seizures from bupropion occur in about 1 in 1000 patients, and it would likely be even higher in a patient with a history of seizures. (4) CONCLUDING: Considering the side effects of both the nicotine patch and bupropion, and his past medical history, it is more likely that the combined therapy would bring on seizures rather than hypertension. (5) RESPONSE: Therefore the patient is at increased risk for seizures, which is not mentioned among the risks in your options (A) Cold intolerance, (B) Hypertension, (C) Polyuria, (D) Renal insufficiency, (E) The patient is at no increased risk. Thus, none of these provided choices would be the greatest risk. The patient's past medical history and symptoms need to be taken into consideration while prescribing medication for smoking cessation.

**Facilitator** **[Round 2]:** Summary: AI_A, AI_B, and AI_C all agree that the greatest risk for the patient using a combination of nicotine patches and bupropion is hypertension. They all reason that both nicotine patches and bupropion can potentially raise blood pressure, especially in a person with a high nicotine dependence. AI_D, however, believes that the greatest risk for the patient is seizures, due to the patient's history of childhood febrile seizures and the fact that bupropion can lower the seizure threshold. AI_D acknowledges that hypertension is a potential risk but does not believe it to be the greatest risk in this case. AI_E, on the other hand, believes that the patient is at no significant increased risk from the combination therapy. AI_E reasons that while every medication carries some risks and potential side effects, the combination of nicotine replacement therapy with bupropion is generally well tolerated and is not typically associated with the specific risks listed. Question to clarify disagreements: Given the patient's history of childhood febrile seizures and the fact that bupropion can lower the seizure threshold, why do AI_A, AI_B, and AI_C not consider seizures as a potential risk? Conversely, why does AI_D not consider hypertension as the greatest risk, given that both nicotine patches and bupropion can potentially raise blood pressure? Lastly, can AI_E elaborate on why they believe the patient is at no significant increased risk, despite the potential side effects of both nicotine patches and bupropion?. Based on your preceding response, how would you answer the original question: [A 28-year-old man comes to the office because he would like to quit smoking cigarettes. He says, ""I cannot go more than a day without cigarettes because I get so anxious and irritable. I even wake up at night to smoke."" The patient began smoking cigarettes at age 12 years; he was smoking two packs of cigarettes daily by age 17 years, and he has not curtailed his use since that time. He does not drink alcoholic beverages or use illicit drugs. Medical history is remarkable for childhood febrile seizures. He takes no medications. Vital signs are normal. The patient is interested in using a nicotine patch and bupropion therapy because he says his friend found the treatments to be helpful. In combining these two therapies, this patient is at greatest risk for which of the following? (A) Cold intolerance (B) Hypertension (C) Polyuria (D) Renal insufficiency (E) The patient is at no increased risk]

**AI_B:** RESTATING THE QUESTION: The inquiry presented explores the potential risks for a 28-year-old male who's aiming to quit smoking using a combination therapy of nicotine patch and bupropion. The possible risk factors to focus on are cold intolerance, hypertension, polyuria, renal insufficiency, or whether there's no significant increase in risk. The patient's notable medical history includes childhood febrile seizures. PLANNING: Primary assumptions to be addressed include the effect of nicotine replacement therapy and bupropion on various physiological systems and the impact of this individual's history of childhood febrile seizures. As alternative assumptions, I propose examining the potential cardiovascular impacts of this therapy on a chronic smoker and considering the neurologic implications of bupropion use in a person with a history of seizures. CLINICAL REASONING: When examining the physiological effects of nicotine and bupropion on the body, one might hypothesize that both can exert stimulatory effects which might increase blood pressure, hence leading to hypertension (Option B). Given the patient's extensive history of smoking, this seems a pertinent risk. The neurologic effect of bupropion, particularly its potential to lower the seizure threshold, could also be a concern considering the patient's history of childhood febrile seizures. However, childhood febrile seizures typically don't predispose to the development of adult-onset epilepsy, and their occurrence in the context of a fever suggests a contextual seizure threshold-lowering factor that isn't necessarily present in this case. CONCLUDING: The cumulative clinical reasoning points toward hypertension as the most significant risk. Any interaction between the patient's history of febrile seizures and bupropion-induced seizure risk doesn't seem substantial as per current knowledge, while the hypertensive effect of the applied therapies in the context of chronic nicotine dependence seems more direct and probable. RESPONSE: From a clinical perspective, considering the patient's history and the physiological effects of nicotine replacement therapy and bupropion, the patient is at greatest risk for hypertension (Option B).

**AI_C:** RESTATING THE QUESTION: The query wants us to determine which risk factor is most probable for this 28-year-old man with a high nicotine dependency, considering he is planning on combining nicotine patches and a bupropion therapy to help curb his smoking habit. Using my knowledge in various domains, such as pharmacology and pathology, I can understand that this is a case of managing nicotine withdrawal symptoms with therapy. PLANNING: Assuming that both nicotine patches and bupropion therapy have side effects, we could guess that this patient is at risk of one or more of the listed complications which include: cold intolerance, hypertension, polyuria, or renal insufficiency. Alternatively, assuming that his medical history and current health status could play a part, we could question if the man's history of childhood febrile seizures or his chronic smoking could heighten certain risks. One critical assumption to evaluate is the risk of hypertension from the combination of nicotine patches and bupropion therapy, especially in an individual with high nicotine dependence. CLINICAL REASONING: Nicotine, whether from cigarettes or therapeutic patches, is a known stimulant that can cause transient increases in blood pressure and heart rate. Bupropion, on the other hand, is a norepinephrine-dopamine reuptake inhibitor; it can also raise blood pressure as a side effect. Thus, the combination therapy in a high nicotine-dependent individual could potentially boost the risk of hypertension. However, when considering the seizures aspect, despite bupropion’s potential to lower the seizure threshold, the patient's childhood febrile seizures do not inherently elevate his risk for non-febrile seizures in adulthood. CONCLUDING: While other risks are possible and should not be ignored, it is clinically prudent to focus on managing the potential risk of hypertension due to chronic nicotine dependence and the stimulating effects of the proposed therapies. Therefore, regular monitoring of the patient's blood pressure during therapy should be part of the management plan. RESPONSE: Hence, with all factors considered, it would seem that amongst the given options, this patient is at greatest risk for hypertension (Option B) when combining nicotine patch and bupropion therapy for smoking cessation.

**AI_D:** Restating the question: The scenario presents a 28-year-old male who is a chronic smoker seeking to quit using a nicotine patch and bupropion. Since he has a history of childhood febrile seizures, we're being asked to assess the potential risks associated with his planned therapeutic interventions, specifically from choices (A) Cold intolerance, (B) Hypertension, (C) Polyuria, (D) Renal insufficiency and (E) The patient is at no increased risk. Planning: In order to determine which associated risks could be of greatest importance here, we must keep in mind the pharmacology and possible side effects of the nicotine patch and bupropion, as well as the patient's medical history. Nicotine substitutes may increase the risk of hypertension due to nicotine’s stimulating effect on the sympathetic nervous system. Bupropion, an atypical antidepressant, can potentially lead to seizures, particularly in individuals with a history of seizure disorders. Clinical Reasoning: Although hypertension is a common risk factor associated with nicotine substitutes, the patient has no prior history of hypertension. The concern should shift towards the risk of seizures due to bupropion's potential to lower the seizure threshold, factoring in the patient's history of childhood febrile seizures. However, it must be stated that febrile seizures in childhood do not necessarily predispose an individual to seizures in adulthood, but caution must still be taken. Concluding: While the patient may indeed be at a minor risk of hypertension from the dual therapy due to the effects of both the nicotine patch and bupropion, the most significant risk from the options provided would be seizures from bupropion given the history of febrile seizures in childhood. However, it is crucial to monitor both parameters in the patient. Response: The given scenario doesn't provide the option of seizures, which would be the significant risk in this patient due to the use of bupropion. The choices offered do not fully align with the clinical reasoning explored above. Therefore, from the given choices, the patient would be at a greater risk for (B) Hypertension. It's significant to note that the patient should be adequately counselled about the potential risks associated with the use of bupropion particularly seizures given his history of febrile seizures.

**AI_A:** RESTATING THE QUESTION: The query seeks to understand which potential risk this 28-year-old male, a long-time smoker who wants to quit by using a nicotine patch and bupropion, faces the most. His medical history involves childhood febrile seizures, and he's a regular, heavy smoker. It is important to note that the nicotine patch is a form of nicotine replacement therapy and bupropion is an atypical antidepressant and smoking cessation aid. The options provided range from cold intolerance, hypertension, polyuria, renal insufficiency or deem the patient is at no increased risk. PLANNING: Considering the effects of the therapies the patient is interested in, and keeping his medical history in mind, it seems crucial to note that bupropion can, in some cases, lead to hypertension. Also, both bupropion and nicotine are CNS stimulants - they might increase systemic vascular resistance, leading to systemic hypertension. Thus, it might seem the patient could be at risk of hypertension. However, his history of febrile seizures raises another point: bupropion can lower the seizure threshold, potentially increasing seizure risk in predisposed individuals. Yet, the question also includes potential risks like cold intolerance, polyuria, and renal insufficiency, or the possibility that the patient is at no increased risk, all of which would demand a well-thought clinical approach. CLINICAL REASONING: Regarding cold intolerance, neither the nicotine patch nor bupropion is known to cause this. The risk of polyuria is particularly associated with the use of certain drugs like diuretics and not typically with bupropion or nicotine patches, making it less likely here. Renal insufficiency isn't a known side effect or risk of either medication and there's no information suggesting renal issues in the patient's medical history, eliminating this option. Upon examination of the patient's history and the properties of the suggested therapies, the risk of hypertension appears most plausible, given both nicotine patch and bupropion could potentially increase blood pressure. However, it's also important to consider the patient's past experience with seizures. Bupropion can decrease the seizure threshold, potentially making the patient prone to them. Nonetheless, it's noteworthy that his seizures were febrile seizures during childhood, which typically do not continue into adulthood. CONCLUDING: After meticulously connecting the dots, given the patient's medical history and the properties of the therapies he is considering, the risk seems skewed towards hypertension. Although there is a potential risk of seizures due to bupropion's effect on the seizure threshold, his childhood febrile seizures are less likely to play a significant role in adulthood. Cold intolerance, polyuria, and renal insufficiency do not emerge as strong contenders in this context. RESPONSE: Thus, this patient, looking to combine a nicotine patch and bupropion therapy, appears to be at greater risk for hypertension (B). While some risks like seizures cannot be entirely ruled out, their probability is relatively low in comparison to the potential of developing hypertension considering the pharmacological characteristics of the treatments in question.

**AI_E:** 1) RESTATING THE QUESTION: This patient, a 28-year-old man with a history of heavy smoking and childhood febrile seizures, is interested in pursuing nicotine patch and bupropion therapy to aid in his smoking cessation efforts. He's asking about potential risks associated with this combination therapy. The term "bupropion" refers to a type of medication classified as a dopamine/norepinephrine reuptake inhibitor used in smoking cessation. 2) PLANNING: Considering his history, one might initially suspect the risk lies in dealing with potential withdrawal symptoms or possible provocation of a seizure, given his childhood history. However, it's also important to consider the effects of nicotine patches and bupropion. Both can potentially elevate blood pressure, and bupropion can decrease seizure threshold. Therefore, hypertension and seizures are the clinically prioritized risks to pursue. 3) CLINICAL REASONING: The first step in clinical reasoning involves considering the patient's smoking history. Given his heavy and prolonged use, he likely has developed high nicotine dependence. The withdrawal symptoms he describes (anxiety, irritability) further support this. When treating such patients, medications like nicotine patches (which replace some of the nicotine from cigarettes) and bupropion (which reduce cravings and withdrawal symptoms) can be effective. The nicotine patch, however, causes a continuous delivery of nicotine which may lead to an increased systemic load of nicotine compared with smoking, causing an elevating effect on blood pressure. Similarly, bupropion can also cause an increase in blood pressure but not necessarily in all patients. This warrants monitoring of blood pressure, especially in the initial weeks of therapy, and even more so in patients with a prior history of hypertension which our patient does not have. The role of bupropion in lowering the seizure threshold is indeed a valid concern, but it should be noted that the patient's febrile seizures were during childhood and many outgrow these seizures with no recurrence in adulthood. Furthermore, the risk of seizures with bupropion is more prominent when high doses are used and in patients with predisposing conditions like eating disorders, uncontrolled epilepsy, or abrupt discontinuation of alcohol or benzodiazepines which our patient does not seem to have. 4) CONCLUDING: With consideration to the clinical reasoning, it can be concluded that while using a combination of nicotine patches and bupropion therapy, the most significant risk to our patient could be the potential development of hypertension due to the additive hypertensive effects of both therapies. 5) RESPONSE: Thus, considering the patient's unique circumstances and the combined effects of these therapies, the patient is at greatest risk for hypertension (B) due to the potential hyperadrenergic state induced by this combined therapy. Contrarily, given the absence of a personal history of recurrent seizures or conditions predisposing to seizures, the risk of seizures is considered less significant in this profile. As such, while the patient will require monitoring of blood pressure while using these two therapies, the choice to use a combination of nicotine patches and bupropion remains a well-tolerated and generally safe strategy for smoking cessation in his case, given the current information.

**Facilitator** The consensus statement synthesizes the responses of AI_A, AI_B, AI_C, AI_D, and AI_E as follows: The question presents a scenario of a 28-year-old male, a chronic smoker, who is considering using a nicotine patch and bupropion therapy to quit smoking. His medical history includes childhood febrile seizures. The potential risks associated with his planned therapeutic interventions are cold intolerance, hypertension, polyuria, renal insufficiency, or no increased risk. Both nicotine patches and bupropion therapy have potential side effects. Nicotine, whether from cigarettes or therapeutic patches, is a known stimulant that can cause transient increases in blood pressure and heart rate. Bupropion, a norepinephrine-dopamine reuptake inhibitor, can also raise blood pressure as a side effect. Thus, the combination therapy in a high nicotine-dependent individual could potentially boost the risk of hypertension. However, bupropion also has the potential to lower the seizure threshold, which could be a concern considering the patient's history of childhood febrile seizures. Nevertheless, childhood febrile seizures typically do not predispose to the development of adult-onset epilepsy, and their occurrence in the context of a fever suggests a contextual seizure threshold-lowering factor that isn't necessarily present in this case. Cold intolerance, polyuria, and renal insufficiency are not typically associated with the use of nicotine patches or bupropion, making these risks less likely. In conclusion, considering the patient's history and the physiological effects of nicotine replacement therapy and bupropion, the patient is at greatest risk for hypertension. While the risk of seizures cannot be entirely ruled out, their probability is relatively low in comparison to the potential of developing hypertension considering the pharmacological characteristics of the treatments in question. Therefore, regular monitoring of the patient's blood pressure during therapy should be part of the management plan. It's also significant to note that the patient should be adequately counselled about the potential risks associated with the use of bupropion, particularly seizures, given his history of febrile seizures.
